# Obituary: Haig Kazazian and Horizontal Transfer (1937 – 2022)

**DOI:** 10.1186/s13100-022-00286-y

**Published:** 2022-12-28

**Authors:** Mobile DNA Editorial Board

**Affiliations:** The Campus, 4 Crinan Street, London, UK

**Figure:**
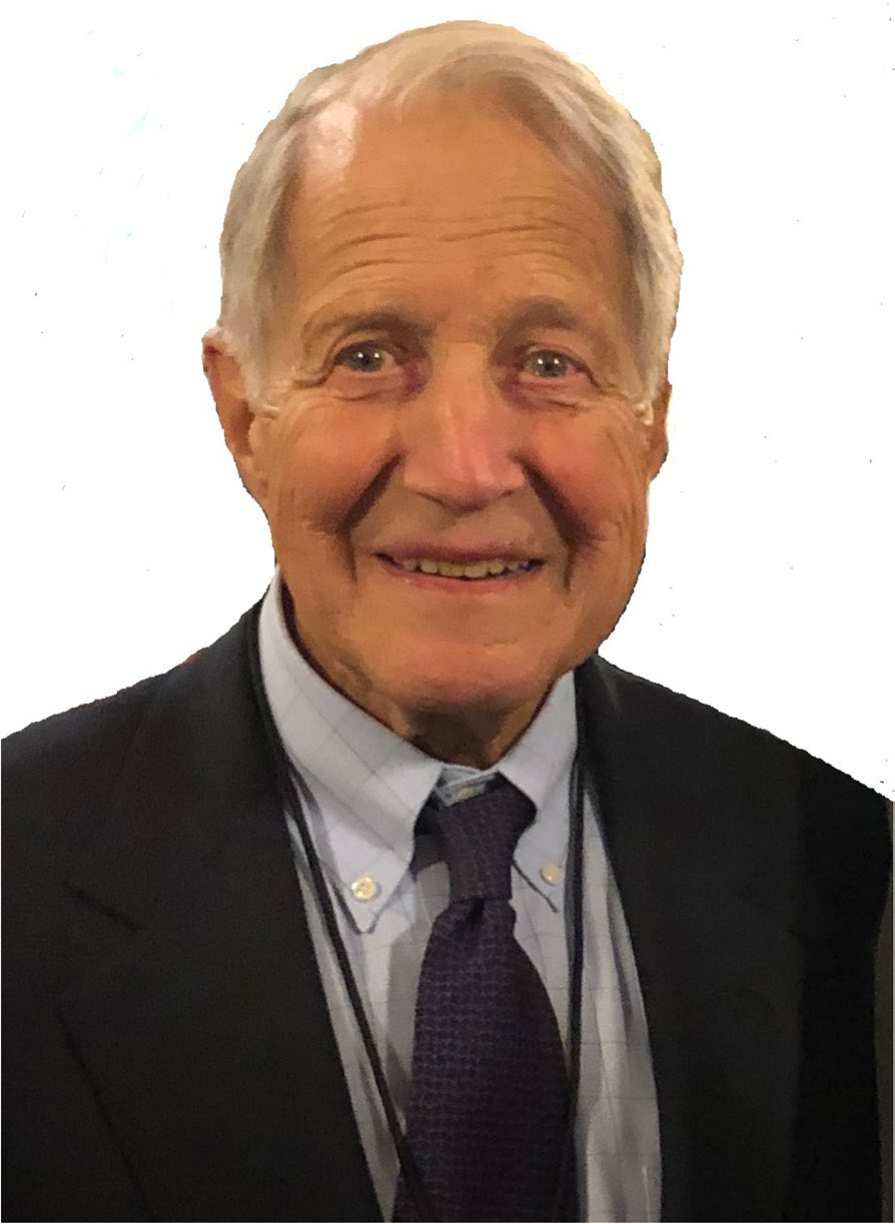
**Haig Kazazian (1937 – 2022)**

In January the Mobile DNA field lost one of its most influential investigators, Dr. Haig Kazazian, the physician-scientist who first demonstrated the activity of transposable elements in humans and whose laboratories at Johns Hopkins University School of Medicine and the University of Pennsylvania made outsized contributions to our understandings of long interspersed element-1 (LINE-1). Several articles and meetings have chronicled Haig’s career and honored his impact on transposable element biology and human genetics. For *Mobile DNA*, where Haig served as Editor-in-Chief, we decided to provide a place for his former lab members to reflect on what it was like to train with him. Their responses capture perspectives on Haig as a mentor – an inviting and brilliant-minded scientist whose delight in the next discovery was catching. All of us who had the pleasure and privilege of discussing science regularly with Haig remember leaving these conversations filled with wonder and energy.

LINE-1 transmits vertically.

But for me, recalling Haig conjures thoughts of horizontal transfer.

Kathleen H. Burns, M.D., Ph.D.

*I met Haig soon after he arrived at Penn. I was an M.D., Ph.D. student and was randomly (luckily!) assigned to present an article Haig had chosen for journal club. It was Tom Eickbush’s classic Cell paper describing how nicks at the site of insertion prime reverse transcription in R2Bm. I was hooked on retrotransposons right away, so I asked to join Haig’s lab for my thesis. Instead of agreeing, Haig invited me out for pizza. We talked about science, but also about our families and other interests. After dinner, Haig said, “Sure, you can join the lab.” I didn’t understand at the time, but of course Haig was making sure I would fit in with the environment he had cultivated in his incredible lab. (… It’s good that I didn’t accidentally say anything negative about the Orioles ...)*

*Over the next 4 years, and 25 more after that, Haig taught me so much about being a scientist. He taught me about rigor and being critical of your own work. He also taught me to take pride in teaching and mentoring. What an incredible mentor Haig was. It’s one thing to support trainees when they work for you. It’s quite another to treasure their success as much as your own for long after that. Haig never stopped mentoring me. Calling to check up, nominating me for positions and awards, and listening whenever I needed a sounding board. His generosity and enthusiasm were boundless.*

*We call ourselves the scientific “children” of our mentors, and Haig’s long career gave his scientific “children” a large extended family. But calling Haig a scientific “father” is not just a metaphor. He bestowed upon his trainees the lessons any good parent tries to teach their children: the value of curiosity, discipline, persistence, and teamwork. How to be a good citizen of your institution and the broader community. These are the lessons I try to teach my trainees, Haig’s scientific “grandchildren.” Thank you, Haig for your kindness and wisdom - we will miss you.*

**Ralph J. DeBerardinis, M.D., Ph.D.**

**Graduate student, 1994-1998**

*Professor, Children’s Medical Center Research Institute, UT Southwestern Medical Center, Dallas, TX, USA*

*Haig’s work on transposable elements was of immediate interest to me when I was studying intron mobility in yeast mitochondria as a graduate student. His laboratory discovered that LINE-1 insertions could lead to sporadic cases of Hemophilia A and subsequently isolated a potentially active LINE-1. It was apparent that the mobility mechanisms of group II introns and LINE-1 shared similarities, and on the advice of my graduate mentor, Dr. Philip Perlman, I sought postdoctoral training in Haig’s lab. We developed a systematic approach to study LINE-1 mobility in cultured cells, which has been used for almost 25 years to gain important insights into LINE-1 biology, genome evolution, and human disease.*

*Haig leaves us with a legacy of exceptional scientific achievements. Haig also was an outstanding mentor and trained an impressive cohort of independent scientists; I was fortunate to reap the benefits of his tutelage. Haig had many admirable characteristics. He was an eternal optimist, had unquestionable integrity, was a strong proponent of basic science research, and treated people with respect—he made everyone feel “special.” In the lab, Haig combined both hands-off and hands-on mentoring approaches. He gave us space to develop our ideas, but “rounded” in the morning and afternoon to get progress updates. I valued the scientific freedom and found the impromptu check-in meetings valuable, as they helped prioritize what was most important at the time.*

*Haig often remarked, “you had to burn” to do basic research, and looked for that quality when selecting trainees. He valued controls (the more the better), clear logic and thinking, and concise writing skills. Haig was appropriately critical and the lab training environment, at times, could be intense and competitive in a good way, which pushed us to become the best scientists we could be. Haig was very supportive and insisted his trainees present their research at prominent scientific meetings. He took great pride in his trainees’ accomplishments. Looking back, he must have been gratified that many in our cohort achieved success in our chosen fields.*

*Haig was a consummate teacher regaling us with detailed stories recalling key discoveries in human genetics, in a flourished fashion, that stuck in your memory. Training with him made me realize that people remember stories more than lists of facts; I apply what I learned from Haig in my own lectures. Simply stated, much of what I know about human genetics was fueled by Haig’s knowledge of the field.*

*Haig and I spoke about once a week for over 20 years. Our conversations focused on science, the latest findings in the field, baseball (I am a Yankees fan; Haig, an Orioles fan), and, more frequently over time, family. Haig loved his family and was extremely proud of his wife, Lilli, his children, and grandchildren. Haig often asked about my family, celebrated his trainees’ successes, and, most importantly, was always there for me (and undoubtedly his trainees) when I faced unexpected setbacks and career challenges.*

*Upon hearing the news of Haig’s passing, some colleagues at Michigan remarked that I was fortunate to have a close relationship with Haig. They are right. Haig welcomed me into the field and remained my mentor for almost 30 years; our mentor/mentee relationship evolved into a close friendship. I will continue to do my best to pay forward the many lessons I learned from Haig to my own trainees. Haig was a luminary in the field of human genetics and inspired a new generation of scientists to follow in his footsteps. He will always be remembered.*

**John V. Moran, Ph.D.**

**Postdoctoral Fellow, 1994-1998**

*Professor, Department of Human Genetics, University of Michigan Medical School, Ann Arbor, MI, USA*

*It is so hard to put into words because you are flooded with so many fond memories along with many hilarious moments. I can almost hear him walking down the hallway with his buoyant and ever cheerful gait. Haig was simply brilliant and full of optimism. His lab meetings were so relaxed and yet you learnt so much. He had an amazing leadership style with absolute academic freedom that brought out the best in each of us. I have never seen him upset about anything in all the years that I have known him. If experiments did not work, he was never down for he genuinely believed that it would work out next time. He loved his lab and his family. Haig’s life was a dream we all wished for. To quote his own words – “a charmed life, you get all that you wished for”. He will be dearly missed. I am so fortunate to have been a part of his great academic family.*

**Rita Sarkar, Ph.D.**

**Postdoctoral fellow and faculty, 1994-2004**

*Program Director, National Heart, Lung and Blood Institute, National Institutes of Health, Bethesda, MD, USA*

*I did my Ph.D. thesis in Molecular Biology at the University of Pennsylvania (UPenn) in Haig’s laboratory. I had the pleasure of having him come out to Poseida Therapeutics to give a talk about his career where I rightfully introduced him as a national treasure. His scientific career was remarkable in both duration and productivity. Haig’s first publication as a medical student was in Nature. Not a bad start - and his success never let up with ultimately many hundreds of publications in top journals. Haig had seminal contributions to the field of genetics, including in the areas of X chromosome inactivation, haplotype analysis and analysis of genetic mutations in various hemoglobinopathies. One of the mutations he discovered in a child with hemophilia A was caused by an active LINE-1 retrotransposon.*

*I met Haig in 1995 when I was a first-year medical student at UPenn. At the time, half of his laboratory was working on gene therapy for hemophilia A and the other half was working on the LINE-1 retrotransposon. I was the first student in the gene therapy program at UPenn and decided to do my Ph.D. thesis in his laboratory focused on studying the mechanism of LINE-1 retrotransposition, but with an eye towards using the retrotransposon as a gene therapy tool to cure genetic diseases. Over two decades later, we at Poseida are planning to enter the clinic with a product candidate called P-FVIII-101, which we believe could be a single treatment cure for hemophilia A. We use a transposable element called piggyBac as part of our therapy. It is quite ironic that Haig’s lab discovered an active LINE-1 retrotransposon as the cause of hemophilia A in a patient, and now we are using another transposable element to try to cure the disease for patients living with hemophilia A!*

*I once asked Haig what he thought of as the greatest discovery in genetics during his career. His answer was the discovery of restriction nucleases because “there wasn’t much you could do in molecular biology before then.” That seems like an understatement! I suppose I might answer “site-specific nucleases” if someone asked me the same question. It is also notable that Haig’s lab was a “beta tester” for polymerase chain reaction (PCR) using Taq polymerase when that was discovered. Before the introduction of Taq he said that you had to cycle the PCR reaction by moving tubes to different temperature water baths and that was “laborious.” Another understatement!*

*Haig was a great mentor to me and to many dozens of other Ph.D. students, post-docs, clinical fellows and others. Many of his former students are now themselves doing cutting-edge genetic research at top academic institutes and biotech companies. Haig’s enthusiasm was infectious. Despite running an academic department he made his way to his laboratory at least once and usually several times per day. He would sit and listen to the most recent results produced by every single person in his lab. He was so excited to hear about every experiment – it was like watching a kid in a candy store. I also have to give credit to Haig for giving me a lot of autonomy and the necessary patience to go with it.*

*In addition to being a great mentor, Haig became a great friend. While I was in his lab and after I graduated, Haig would often go to the gym with me and one or two other students. While Haig was never outwardly competitive, he was a high achiever in everything that he did, including lifting weights. Despite the fact that he was in his 60s at the time, Haig would often lift more weight than many of the 20-somethings in the UPenn gym and I could tell that made him pretty pleased. After I moved from Philadelphia, I was no longer able to have workouts with Haig, but it was a habit that continued over the decades because every time I visited Haig or he visited me he always wanted to go to the gym. To this day my workouts with Haig are among my fondest memories. Haig was an elegant gentleman, a renowned scientist, a phenomenal mentor and a true friend to me and many others. The world is a lesser place without him.*

**Eric Ostertag, M.D., Ph.D.**

**Graduate student, 1997-2001**

*Executive Chairman, Poseida Therapeutics, Inc., San Diego, CA, USA*

*Haig loved all things genome. So often, with obvious joy on his face, he sat side-by-side with students reading out sequence - CGTATTCCTCATCACAAG, and so on -- a joy perhaps dampened somewhat by the advent of Illumina sequencing. Nevertheless, Haig was pleased the new technologies drew many new colleagues into the mobile DNA field. The epitome of collegiality, Haig enjoyed interacting with all. On the other hand, sloppy thinking was little condoned and the guilty would politely but firmly be set right. This would occasionally manifest as whispered and running asides during scientific talks -- although, usually Haig sat near the front, and I sat near the back.*

*I will miss Haig’s enthusiasm. I will miss his insight. I will miss his joie de vivre. I will miss the enormous box of baklava that Haig ordered for his group every Christmas, and the conviviality we enjoyed eating it together, while standing in the lab.*

*Ave atque vale Haig.*

**John Goodier, M.Sc., Ph.D.**

**Postdoctoral fellow and faculty, 1998-2020**

*Johns Hopkins School of Medicine (retired)*

*Haig is a great man who lived a long healthy full life. He rescued my PhD, but I’ll always remember him whole heartedly joining us for lifting, his body pump classes and eventually him doggedly curling more weight in the gym with his surprisingly large biceps than I could.*

*He was so disarmingly friendly that it was a year before I realized how bright he was. His ability to quantify phenomena was unlike any I’ve ever seen. He could always find some extra novelty or number to make your science more interesting.*

*He advised me to keep my relationship with my wife and family strong and simple. “You can’t be productive if your life at home isn’t peaceful.”*

*Here’s to Haig!! I’ll miss him, but will take comfort in the fact that he lived an enviable life. He did it right and will continue to serve as one of my top role models.*

**Brook Brouha, M.D., Ph.D.**

**Graduate student, 1999-2003**

*Dermatiologist, West Dermatology, La Jolla, CA, USA*

*Haig was a great mentor for so many, including myself during my postdoctoral time in his research group at the University of Pennsylvania, and after I had relocated back to my home country to start my own research group. There are numerous rememberable things. Haig was a very kind, generous, humble person, very respectful towards others. Haig’s genuine interest in research is among the most memorable. If Haig’s schedule permitted, he would come to the lab at least once per day and talk to lab members about their latest experimental results or progress, or no progress. It was obvious that Haig was truly interested in each members’ science and excited to hear about, and discuss, novel results, even if they were minor. And he clearly wanted to comprehend findings and implications. He would also attach great importance to quality of research and experimentally sound results. He had to be convinced of novel findings, too. Even after I had left the lab, he was very much interested in hearing about novel developments and research findings from my side, and he would be very supportive in various matters over the years.*

*On the other side, he knew there was also a life outside the lab. For example, he did not take it amiss when one day I and colleagues took a day off to go fossil hunting, rather he was very much interested in our finds afterwards. And for the same reason, I have fond memories of Haig, when he had invited us to a Phillies game, explaining to me why a certain company put their advertisement in a certain spot of the ballpark. Indeed, there are so many bigger and smaller memorable things!*

*Haig was a very likeable person, a great scientist who was truly and deeply enthusiastic about doing research and producing novel research findings, and his enthusiasm never ceased. He set an outstanding example of how science should be done and researchers should be mentored. I am very glad I became friends with Haig. I will miss him very much.*

**Jens Mayer, Prof. Dr. rer. nat.**

**Postdoctoral fellow, 1999-2001**

*Professor, Institute of Human Genetics, University of Saarland Medical Faculty, Saarbrücken, Germany*

*As words cannot translate the sorrow of such a tremendous loss, I would just like to express with immense gratitude to Haig how privileged I feel for having known him and been a part of his team. Aside from his great scientific mentorship, I treasure the memory of his exceptional human and caring personality; contagious enthusiasm and optimism, and his frank warm smile. A special dear memory comes to my mind: how genuinely he enjoyed our Wednesday morning lab meetings, which he made so convivial enthusiastically discussing experiments around bagels and cream cheese without fail.*

**Marie del-Carmen Seleme, Ph.D.**

**Postdoctoral fellow, 2000-2006**

*Scientist, Raymond G. Perelman Center for Cellular and Molecular, Therapeutics, Children’s Hospital of Philadelphia, Philadelphia, MD, USA*

*My appreciation of Haig’s work began in 1991, when I read the Science commentary “A “Jumping Gene“ Caught in the Act”. It had been published shortly after a departmental journal club on retrotransposons and was burned into my memory. Around that same time, I was headed back to MS3 rotations, and eventually developed a passion for medical genetics. The years quickly passed during my pediatric training, and I soon started my medical genetics fellowship interviews. I was drawn to the great program at the Children’s Hospital Philadelphia and interviewed with Haig when I visited. At the time, he was the fellowship director, as well as Chair of Genetics. My interviews ended a few weeks after my visit to Penn and I had decided to move to the NIH. I only had to fax the signed offer letter and it was going to be official. The very day I was going to accept the position, at a rare moment when I was home between ER shifts, I received a call …. it was Haig wondering whether I was going to join the CHOP/UPenn program. Later that evening I told my wife we were moving to Philadelphia, not Bethesda, in July. Haig was indeed persuasive in the very best way.*

*I arrived as a clinical and biochemical genetics fellow in the fall of 1999 and became immersed with newborn screening. During this time, I became interested in the disorder methylmalonic acidemia (MMA) because I believed it was relatively common and impossible to treat with conventional medical management. I began to survey possible research interests focused on MMA, with the eventual goal of developing a gene therapy to treat the infants and children with this devastating inborn error of metabolism. To my surprise, the reception for this project by the senior faculty at CHOP and UPENN was disappointing. In fact, no one was interested – “MMA is not a treatable disorder” I was assured. I met with Haig later that fall to review my career plans, evolving interest in MMA, and how we could develop treatments for this disorder if we could develop a research program. At the end of the conversation, Haig proclaimed, “this is a good idea”, which was followed by* “*I’m going to give you space in my laboratory and help you get started on this project”.*

*Haig helped me write my first NIH grant, secure funding, and launch my career. He fully embraced my independence and constantly rose to endorse me, for many years after I left his lab. Haig’s influence has extended not only to me, but to a population of patients with a severe and life-threatening metabolic disorder who now have hope for a more normal life. During my last conversation with Haig in December 2021, I told him that 2 new INDs for MMA had been issued, with a 3rd just submitted (and subsequently approved in March 2022).*

*I think Haig would be proud to know that his last student, a recent JHU undergraduate who was co-mentored by John Goodier, joined my lab in July. The chain of his legacy will continue, and I remain grateful to have received his guidance, support, friendship, and mentorship over the past 20 years.*

**Charles P. Venditti M.D., Ph.D.**

**Clinical fellow, 2000-2003**

*Senior Investigator, National Human Genome Research Institute, National Institutes of Health, Bethesda, MD, USA*

*I joined Haig’s lab as an M.D., Ph.D. student in 2002. At that time, the lab had two main research directions: gene therapy for hemophilia and the biology of mammalian retrotransposons, which became the focus of my work in Haig’s lab. Haig may well have been the only Department Chair at Penn who was in the lab daily, often twice a day, stopping at each lab bench to discuss that day’s results and talk science. His enthusiasm was infectious, as was his pure joy at seeing every new piece of data, every sequence, and every gel. I can picture Haig now drawing the retrotransposition cassette on the blackboard, explaining the mechanisms of LINE-1 inversion, or doing the back-of-the-envelope calculation of retrotransposition frequencies in germ cells. At lunchtime, he sometimes joined students and staff in the modest lunch area at the end of the hall, taking a sincere interest in even the most junior students and sharing generously of himself. We heard stories of his own training and early research experiences, that started with “giant jars of fruit flies” and led to major discoveries, inspiring many of us to follow in his footsteps. Well into his 60s, Haig was a part of the twice-weekly workout group, lifting weights with medical students at the gym after work. I kept in touch with Haig over the years and visited his lab at Hopkins after he moved back to Baltimore. More recently, we caught up during his visits to Penn, exchanging news--mine-about starting my own lab now, and his-about his lab still running strong: “We are now using single-cell technologies to look at somatic L1 insertions!”, “I just received my R01 renewal, now over 30 years of uninterrupted NIH funding!”. Long ago Haig rhetorically asked us: “Do you know what a good project is? It is something that may be hard to do, but, if it were to succeed, it would be really impactful!”. Haig lived by that. Haig was a giant of human genetics, with a spring in his step and unwavering optimism. He will be dearly missed.*

**Daria Babushok, M.D., Ph.D.**

**Graduate student, 2002-2007**

*Assistant Professor of Medicine, Hospital of the University of Pennsylvania, Philadelphia, PA, USA*

*My career in biomedical research really started with Haig, working among the great people in Haig’s lab at the University of Pennsylvania, and later at Johns Hopkins. Haig was an impeccable scientist, always more than happy to talk about ideas and tell you what he really thought. Throughout his career he openly embraced new technologies as they developed - from PCR to next-generation sequencing - with brilliant results driving transposable element research forwards. I am especially grateful to Haig for the many introductions and connections he helped me make throughout the genetics and genomics community as he was widely respected by many.*

*He clearly cared deeply about the career directions of his trainees and did what he could to support them. His mentorship shows in the long list of highly successful scientists trained in his lab spanning diverse career paths. Even more so than science, Haig cared deeply about his family, and if results in the lab weren’t looking good you could always ask him how his grandchildren were doing instead. Haig will be missed by many but his legacy will live on through his trainees and his contributions to Human Genetics and the field of Mobile DNA.*

**Adam Ewing, Ph.D.**

**Graduate student and Postdoctoral fellow 2006-2011**

*Group Leader, Mater Research Institute, University of Queensland, Brisbane, Australia*

*My memories of Haig are grouped into “Haig stories” - which may stem from my own experiences as well as his lore that was passed down among people - and “Haig’s stories.”*

*An example of a “Haig story” that came to mind as I reflected on what I should write is a story that was passed on to me. It was well-known by many that Haig enjoyed the gym. While I was a Ph.D. student at Penn, someone told the story that they once saw Haig in the gym curling the stacks of his reprints from his numerous Nature and Science articles. Silly sure, but the lab had stacks of these reprints. Haig’s output and the quality of that output was remarkable. To this day, I still smile when I remember hearing this.*

*Haig’s stories on the other hand are related to his first-hand telling of not only his science experiences, but also the science that he witnessed and/or was connected to in some way. Being a fan of history, I always really enjoyed these moments; many of which came when Haig would join Adam Ewing and I for lunch at the end of the hall. There are many stories from being on a board of advisors when Mullis pitched the PCR machine to the discussions with Semenza about setting up his system to discover HIF1. Perhaps naturally, my favorites involved him explaining the buildup and discovery of the first* de novo *LINE-1 insertion. There were many dimensions to this story starting with the hypothesis developed early on in genetics that Hemophilia A mutations would largely be* de novo *due to their deleterious nature. This made Hemophilia A an ideal model to understand and characterize disease-causing mutations. The story ending though is what I feel captures his enthusiasm, foresight, and essence - Haig often recounted how he knew immediately once they found that insertion that is what his lab was going to study moving forward. For many of us, the rest is history and part of our story. Haig was a giant that made a positive impact on the world around him and will be missed by many.*

**Dustin Hancks, Ph.D.**

**Graduate student, 2006-2012**

*Assistant Professor, Department of Immunology, UT Southwestern Medical Center, Dallas, TX, USA*

*I worked with Haig as a research fellow and feel extremely lucky to have trained in his laboratory. To me, he was just an encyclopedia of mobile DNA and a generous mentor with piercing intelligence. Haig’s way of conversation with a smiling face was just amazing; I hardly found a second one with the same character. In my 7 years of staying in his laboratory, I never saw him angry with anyone in the lab. Haig was always available to discuss science with you in person or by email. He always looked at experimental data very carefully, and when something was positive, his happiness was inexplicable. He started coming again and again to my workbench and asked many questions before giving me a green light; yes – what I was seeing was true. I love his description of a “ smoking gun” when we had some telling experimental result. When I returned to India, having worked with Haig opened the door to my job, and he gave me an excellent recommendation. He helped me build my laboratory. He was curious about my work, and if I needed to discuss something, he was always available. Haig was also so proud of his family. He always stressed how important it is to find the right partner. He will always be remembered for his enthusiasm for science and his kind attention to the problems of others. Those who had the privilege to work at Haig’s lab over the years will remember him as a father figure and friend.*

**Prabhat Mandal, Ph.D.**

**Postdoctoral fellow, 2006-2013**

*Associate Professor and Group Leader, Dept. of Biosciences and Bioengineering, Indian Institute of Technology, Roorkee, India*

*Haig was always kind and unusually perceptive. I think he saw those of us who worked in his group very well, perhaps better than we saw ourselves at the time. I am sorry that I will no longer run into him spontaneously at ASHG or another conference, or think of him when visiting Baltimore or West Philadelphia. I take inspiration from Haig’s full life, and how very many people he touched.*

**Sanjida Rangwala, Ph.D.**

**Postdoctoral fellow, 2007-2010**

*Staff Scientist, National Center for Biotechnology Information, National Institutes of Health, Silver Spring, MD, USA*

*Haig’s enthusiasm for science was unparalleled in my experience. He would come into the lab at least four to five times per day to chat and look at data “hot off the presses”. Sanger sequencing data may have been his favorite type of data to look at with me. He was a stickler for identifying target site duplication sequences and I will always be a thorough scientist because of him. He was never afraid to ask a question when he was not sure- he would enthusiastically raise his hand and ask as many questions as he needed to in order to understand. Now when I think about it, I realize it’s ok not to know something, but it is unacceptable to not try to understand- and I try to act like Haig, and ask as many questions as I need. He was the consummate gentleman scientist, and there will never be anyone else like him. I was so lucky to have had him as my Ph.D. mentor.*

**Tara Doucet-O’Hare, Ph.D.**

**Graduate student, 2010-2015**

*Research Fellow, National Cancer Institute, National Institutes of Health, Bethesda, MD, USA*

*Dr. Haig had a magical personality, a beautiful smile, and a passion for science, and he was a gentle and humble teacher. I feel privileged to have been a part of his team and extremely lucky to have trained in his lab at Johns Hopkins University. Thank you, Dr. Haig, for continually inspiring me to do my best. I will miss him very much.*

**Priya Tripathi, Ph.D.**

**Postdoctoral fellow, 2014-2015**

*Principal Scientist, Esperovax, Plymouth, MI, USA*

*I had the honor of being in Haig’s lab at Johns Hopkins. After my Ph.D. in endocrinology, I went there to characterize an insertion in the promoter region of the androgen receptor gene. With his guidance, we were able to find a LINE-1 insertion altering the expression of this gene, causing androgen insensitivity. This finding broadened the possibilities of genetic mechanisms leading to sex differentiation disturbances and sex reversal. Thanks to Haig, I was introduced to this fascinating world of mobile DNA and was able to meet incredible researchers in this field. All this experience with Haig has changed the course of our research in this exciting field. I am extremely grateful to him for all I have learned from him.*

**Rafael Loch Batista, M.D., Ph.D.**

**Postdoctoral fellow, 2016**

*Medical Assistant/Associated Researcher, Hospital das Clínicas da Universidade de São Paulo, São Paulo, Brazil*

*I first met Haig in 2012 in Xi’an, China. My department hosted the 8th Chinese National Genetic Disease Diagnosis and Prenatal Diagnosis Research Exchange Conference. I was responsible for the reception of Haig and served as his Chinese translator. My first impression was that Haig was a very scholarly and gentle man, nice to everyone he met. Haig presented a report about mobile DNA at the meeting, the topic was about finding treasures in junk DNA. The response from the audience was very warm and many people asked questions about mobile DNA. I was also very interested in transposon research. After Haig returned to the US we still kept in touch. Because I was a genetics doctor in a women’s and children’s hospital, my research focused on abortion and miscarriage. I wondered if retrotransposons might affect fetal survival and communicated with Haig. He strongly supported my idea and asked me to work in his Johns Hopkins lab for a while to learn techniques about retrotransposons. At the end of 2016, I went to his laboratory. Haig was very serious about the data and asked about experiments in detail. Once I made a mistake about the development of twin fetuses, and Haig was very firm in correcting me. I so realized that I must be serious about data and cannot be careless. Haig was very humble: he only let me call him Haig but never Professor. There I also met Drs. John Goodier and Katsumi Yamaguchi, Haig’s Japanese postdoc. I learned a lot from all of them. Haig gave me a copy of his autobiography, Mobile DNA, with his signature on it and I cherish it. I have been working on L1s and miscarriage since then. I will take Haig as my example, and devote my life to Genetics.*

**Chao Lou, M.D.**

**Visiting Scholar, 2016**

*Attending Physician, Department of Genetics, Northwest Women’s and Children’s Hospital, Xi’an, China*

*first came to know Haig from his review in Science in 2004. As a first year graduate student, this prompted me to reflect on my own retrotransposon research, and I ended up applying to Haig’s lab as a postdoctoral fellow. Haig was a great mentor for me. He would come into the lab for discussions at least twice a day. These discussions were very helpful and allowed me to overcome many difficulties in my work on detecting LINE-1 insertions in single cells. Haig also gave me opportunities to teach many people, from junior high school to Master’s students, to write grants, and to present my research at meetings. He had an excellent memory, remembering the details of a great many diseases, publications, and individuals. I think this helped make his work on genetic diseases great, his research communities strong, and his mentorships successful.*

**Katsumi Yamaguchi, Ph.D.**

**Postdoctoral fellow, 2016-2020**

*Postdoctoral fellow, Graduate School and College of Arts and Sciences, The University of Tokyo, Tokyo, Japan*

